# Maternal Stress and Human Milk Antibodies During the COVID-19 Pandemic

**DOI:** 10.3389/fnut.2022.923501

**Published:** 2022-06-30

**Authors:** Hannah G. Juncker, Eliza J. M. Ruhé, Aniko Korosi, Johannes B. van Goudoever, Marit J. van Gils, Britt J. van Keulen

**Affiliations:** ^1^Department of Pediatrics, Emma Children's Hospital, Amsterdam Reproduction & Development Research Institute, Amsterdam UMC, Vrije Universiteit, University of Amsterdam, Amsterdam, Netherlands; ^2^Swammerdam Institute for Life Sciences - Center for Neuroscience, University of Amsterdam, Amsterdam, Netherlands; ^3^Department of Medical Microbiology and Infection Prevention, Amsterdam Infection and Immunity Institute, Amsterdam UMC, University of Amsterdam, Amsterdam, Netherlands

**Keywords:** SARS-CoV-2, stress, COVID-19, lactation, passive immunity, breast milk

## Abstract

**Importance:**

*SARS-CoV-2*-specific antibodies in human milk might protect the breastfed infant against COVID-19. One of the factors that may influence human milk antibodies is psychological stress, which is suggested to be increased in lactating women during the COVID-19 pandemic.

**Objective:**

To determine whether psychological stress is increased in lactating women during the COVID-19 pandemic, and if maternal stress is associated with the level of *SARS-CoV-2*-specific antibodies in human milk.

**Design:**

Population-based prospective cohort study.

**Setting:**

Data collection took place in the Netherlands between October 2020 and February 2021.

**Participants:**

Lactating women living in the Netherlands were eligible to participate in this study. In total, 2310 women were included.

**Exposures:**

Stress exposure during the COVID-19 pandemic was determined using the Perceived Stress Scale (PSS) questionnaire and maternal lifetime stress was determined by the Life Stressor Checklist – revised (LSC-r) questionnaire.

**Main Outcome(s) and Measure(s):**

Stress experience during the COVID-19 pandemic was compared with a pre-pandemic cohort. *SARS-CoV-2*-specific antibodies in human milk were measured using an Enzyme-Linked Immunosorbent Assay (ELISA) with the Spike protein of *SARS-CoV-2*. The association between maternal stress and human milk antibodies was determined using a multiple regression model.

**Results:**

The PSS score of lactating mothers was not increased during the pandemic compared to the PSS score in the prepandemic cohort. Six hundred ninety-one participants had *SARS-CoV-2*-specific antibodies and were included in the regression models to assess the association between maternal stress and human milk antibodies. No association was found between PSS scores and human milk antibodies. In contrast, the LSC-r score was negatively associated with *SARS-CoV-2*-specific IgA in human milk (β = 0.98, 95% CI: 0.96–0.997, *p* = 0.03).

**Conclusions and Relevance:**

Our results suggest that lactating women in the Netherlands did not experience higher stress levels during the COVID-19 pandemic. Breastfed infants of mothers with high chronic stress levels receive lower amounts of antibodies through human milk, which possibly makes them more vulnerable to respiratory infections. This emphasizes the importance of psychological wellbeing during lactation.

## Introduction

COVID-19 usually has a mild course in children; however, young infants are more susceptible to severe disease development, which could be due to an immature immune system (1). Human milk provides additional immunological protection for these infants as it contains multiple immunological components. Human milk antibodies are suggested to play an important role in the protection against respiratory infections (2–5). Antibodies against the *severe acute respiratory syndrome coronavirus* 2 (*SARS-CoV-2*) have been found in human milk after maternal infection and vaccination (6–12). It is very likely that these antibodies play a critical role in protecting the infant against COVID-19. Indeed, breastfeeding in *SARS-CoV-2* positive mothers, protects their infants from developing symptoms of COVID-19 (13). Moreover, although *SARS-CoV-2* RNA has been detected in human milk, replication competent *SARS-CoV-2* has not been isolated and transmission of the virus to the infant through human milk has not been reported (14–18).

Human milk antibody titers are influenced by many different factors, including maternal psychological stress (19–21). However, there is still controversy on the effect of maternal stress on the secretion of immunoglobulin A (IgA), the most abundant antibody in human milk (19, 22–25). Most studies point toward the view that perceived stress reduces IgA in human milk (19). It is important to elucidate this relationship, as it is plausible to assume that maternal stress might be increased during the COVID-19 pandemic. Indeed, several studies have highlighted concerns about the mental health of postpartum women in the COVID-19 pandemic, showing an increase in depressive symptoms, anxiety and maternal distress (26–29). The mental state and overall functioning of the mothers may have suffered from the lockdown measures due to limited access to support systems, changes in hospital policies including unaccompanied pregnancy checkups, mother-infant separation policies, and the stress that comes from their overall concerns about exposure to COVID-19 (29).

The aim of this study is to investigate maternal psychological stress during the COVID-19 pandemic and its potential impact on *SARS-CoV-2*-specific antibodies in human milk. We hypothesize that maternal psychological stress is higher during the pandemic and that perceived stress levels are negatively associated with IgA against *SARS-CoV-2* in human milk.

## Methodology

### Study Design and Population

The COVID MILK – POWER MILK study is a prospective cohort study, which included lactating women between October 12th and February 23th in the Netherlands who did not yet receive a *SARS-CoV-2* vaccine. Participants were recruited via (social) media and could sign themselves up by sending an e-mail. Ethical approval was obtained from the Medical Ethics Committee of the Amsterdam UMC, location VUmc. Written informed consent was obtained from all participants.

### Study Procedures and Sample Collection

To determine *SARS-CoV-2* antibodies, a human milk and blood sample were collected during a study visit. In the morning of the appointment, participants were instructed to empty one breast completely before the first feeding moment, either manually or with an electric breast pump, mix the milk and subsequently store 20 ml in the refrigerator until collection by the researcher. During the study visit, 5 ml of blood was collected. At the study site, serum and milk samples were stored at −80°C up until analysis. After the study visit, participants received a questionnaire, which included two validated test tools to examine the level of stress experienced by the participants.

### Perceived Stress Scale (PSS)

To investigate stress during the COVID-19 pandemic and its influence on maternal antibodies, the PSS questionnaire was used. The PSS is a validated 14-item questionnaire developed by Cohen et al. (30, 31). The questionnaire aims to determine how stressful one experiences certain situations (30, 31). For each question the respondent is asked to indicate how many times they felt a certain way since the outbreak of COVID-19. Each question is scored on a 5-point Likert Scale ranging from 0–4 (0 = never; 1 = almost never; 2 = sometimes; 3 = fairly often; 4 = very often).

### Life Stressor Checklist – Revised (LSC-r)

To investigate the influence of maternal lifetime stressors on human milk antibody levels, the LSC-r questionnaire was used. The LSC-r evaluates the maternal lifetime history of stress. The validated checklist is a 30-item scale to identify the exposure to traumatic events or other stressful life events (32). For this research, we used the questions that form a comparative baseline for lifetime traumatic stress. We combined two scoring methods of the questionnaire for this study. This approach combines a score for high magnitude stressors (criteria A stressors) and a score for low magnitude stressors (other life stressors) (32) resulting in an overall life stressor score ranging from 0–13, with the highest score representing the highest level of lifetime history of stress (32).

### Determination of SARS-CoV-2 Antibody Titers in Human Milk and Serum

Before analysis, the collected human milk and serum samples were stored at the Amsterdam UMC, location VUmc, at −80°C. To assess the *SARS-CoV-2*-specific IgA antibodies in human milk and IgG antibodies in serum, an enzyme-linked immunosorbent assay (ELISA) with the *SARS-CoV-2* spike protein was used as described previously (33). In brief, whole human milk and serum samples were diluted 1:10 or 1:100, respectively, in 1% casein PBS (Thermo Scientific) and IgA or IgG were detected using horseradish peroxidase (HRP)-labeled goat anti-human IgA (Biolegend) or HRP-labeled goat anti-human IgG (Jackson, Immunoresearch), respectively, which were validated using monoclonal antibodies. A relative operating characteristic (ROC) curve analysis was performed to determine the cut-off value for both milk and serum samples using pre-pandemic negative samples and polymerase chain reaction proven positive samples. The human milk samples were considered positive at an optical density (OD) 450 nm cut-off value of 0.502, and the serum samples at an OD450 nm value of 0.452. With these cut-off values, the sensitivity was 67.9% (95% confidence interval (CI): 61.0–74.1%) for IgA antibodies in human milk with a specificity of 99.0% (95% CI: 94.7–100.0%) and for serum IgG antibodies the sensitivity was 95.9 (95% CI: 92.9–97.6%) with a specificity of 99.1 (95% CI: 94.9–100%). For cross-comparison, negative and positive controls were included in each run.

### Statistical Analysis

The obtained data is registered in the Clinical Data Management System “Castor Electronic Data Capture (EDC).” In order to perform the statistical analysis, the data was transferred into IBM Statistical Package for Social Sciences Statistics (SPSS) for Windows version 26. Characteristics were described in descriptive statistics including frequencies, mean values with standard deviations (SD) or median with interquartile ranges (IQR). Participants with missing data for stress measures or antibody levels were excluded from further analyses.

We compared PSS scores in our cohort with a recent study conducted in the United States before the outbreak of COVID-19 (34). This pre-pandemic cohort consisted of 151 lactating mothers between 18 and 40 years old who filled out the PSS questionnaire at weeks 1 and 2 postpartum, as well as at 1-, 2-, 3-, and 6-months postpartum. This pre-pandemic cohort was comparable with our cohort in baseline characteristics including age, BMI and history of depression. Unpaired *t*-tests were performed to compare PSS scores between this pre-pandemic cohort and our cohort for each month postpartum.

To investigate the influence of maternal stress on human milk antibodies, lactating mothers who tested positive for *SARS-CoV-2*-specific antibodies in serum or human milk were included. IgA values were log-transformed before analyses. Due to a non-linear relation between PSS and IgA levels, participants were divided in three groups: low stress (PSS 0-14.99), moderate stress (15.00–21.99) and high stress (22.00–56.00) based on the 33.3–66.6 percentiles. Pearson Chi square tests, one-way ANOVA and Kruskal Wallis tests were used to assess differences in characteristics between PSS subgroups based on the distribution.

To examine the association between PSS and LSC-r scores and maternal antibodies, multiple regression analyses were performed. The PSS regression model was adjusted for factors that differed between the PSS groups. In literature, age of the mother, BMI of the mother, parity, lactation stage and sex of the child have shown to influence antibody levels in human milk (21, 23, 35, 36). Those variables were added to the LSC-r regression model when they influenced the model with >10%. To correct for the logarithmic transformation, the following formulas were used to accurately interpret the regression coefficients: β = *e*^β^ and 95.0% confidence interval= *e*^(β±1.96 *x standard error*)^. For the statistical analysis, the hypothesis was tested two-tailed and a *p*-value of <0.05 was considered statistically relevant. GraphPad Prism for Windows (version 8.2.1.) was used to illustrate the data distributions.

## Results

### Stress Levels of Lactating Mothers During the COVID-19 Pandemic

#### Baseline Characteristics

In total, 2310 mothers participated in the study, of whom 2,163 (94%) filled out the characteristics questionnaire (Table 1). The participants were on average 33.2 (SD ± 3.9) years of age and were breastfeeding their child for 38.0 (25.0–59.0) weeks.

**Table 1:** Participants characteristics based on perceived stress scores (PSS) in participants with an ELISA confirmed SARS-CoV- 2 infection in serum or human milk.

			**Perceived Stress Scale groups**			
**Maternal characteristics**	**Total (*N* = 2,310)**	***SARS-CoV-2* positive (*N* = 691)^**a**^**	**Low (*N* = 182)^**a**^**	**Moderate (*N* = 245)^**a**^**	**High (*N* = 219)^**a**^**	***p*-value**
Age mother – years* (± SD)	33.1 (±3.8) (*N* = 2,223)	33.2 (± 3.9) (*N* = 662)	33.5 (± 3.9) (*N* = 175)	33.0 (± 3.8) (*N* = 240)	33.3 (± 4.1) (*N* = 208)	0.46
Body Mass Index** (IQR)	23.3 (21.3–26.0) (*N* = 2,226)	23.3 (21.4–25.9) (*N* = 646)	23.1 (21.4–25.5) (*N* = 182)	23.0 (21.1–25.8) (*N* = 245)	23.6 (21.−26.2) (*N* = 219)	0.16
Chronic illness No. (%)	306/2,224 (13.8)	81/646 (12.5)	18/182 (10.0)	35/245 (14.3)	28/219 (12.8)	0.41
Autoimmune disease No. (%)	72/2,262 (3.2)	15/646 (2.3)	2/182 (1.1)	7/245 (2.9)	6/219 (2.7)	0.46
Psychological disease No. (%)	408/2,221 (18.4)	106/645 (16.4)	20/182 (11.0)	28/245 (11.4)	58/218 (26.6)	0.0001
Smoking No. (%)	42/2,196 (1.9)	12/636 (1.9)	0/179 (0)	7/242 (2.9)	5/215 (2.3)	0.048
Alcohol consumption No. (%)	1,014/2,196 (46.2)	338/636 (53.1)	96/179 (53.6)	136/242 (56.2)	106/215 (49.3)	0.33
LSC-r score** (IQR)	1.0 (0.0–3.0) (*N* = 2,226)	1.0 (0.0-3.0)	1.0 (0.0–2.0) (*N* = 182)	1.0 (0.0–3.0) (*N* = 245)	2.0 (1.0–4.0) (*N* = 219)	0.0001
**Education level**						0.02
- Primary and lower secondary No. (%)	31/2,263 (1.4)	7/673 (1.0)	3/182 (1.6)	3/245 (1.2)	1/219 (0.4)	
- Upper secondary No. (%)	338/2,263 (14.9)	114/673 (16.9)	22/182 (12.1)	37/245 (15.1)	54/219 (24.6)	
- Bachelor equivalent No. (%)	1,008/2,263 (44.5)	291/673 (43.2)	82/182 (45.1)	99/245 (40.4)	97/219 (44.3)	
- Master and Doctoral equivalent No. (%)	842/2,263 (37.2)	245/673 (36.4)	73/182 (40.1)	103/245 (42.0)	65/219 (29.7)	
**Infant characteristics**						
Age child – weeks** (IQR)	34.0 (24.0–50.0) (*N* = 2,122)	38.0 (25.0–59.0)	37.0 (26.0–56.3) (*N* = 174)	35.0 (23.3–55.0) (*N* = 234)	42.0 (28.0–66.0) (*N* = 210)	0.005
GA at delivery – weeks** (IQR)	40.0 (39.0-40.9) (*N* = 2,164)	40.1 (39.0-40.9)	40.0 (39.0-40.9) (*N* = 176)	40.2 (39.3-41.0) (*N* = 240)	40.0 (39.0–40.9) (*N* = 211)	0.52
Birth Weight – grams* (± SD)	3,566 (±517) (*N* = 2,160)	3,582 (± 517) (*N* = 637)	3,579 (± 510) (*N* = 175)	3,605 (± 517) (*N* = 240)	3,562 (± 532) (*N* = 208)	0.72
Primipara No. (%)	865/2,185 (39.6)	244/635 (38.4)	76/179 (42.5)	92/242 (38.0)	76/214 (35.5)	0.37
Sexe- Boy No. (%)	1,071/2,233 (45.5)	318/635 (50.0)	83/179 (46.4)	122/242 (50.4)	113/214 (52.8)	0.45
**Delivery**						
Vaginal delivery No. (%)	1,835/2,236 (82.1)	532/635 (78.1)	147/179 (82.1)	204/242 (84.3)	181/214 (84.6)	0.79
Instrumental delivery No. (%)	129/2,236 (5.8)	37/635 (5.8)	14/179 (7.8)	13/242 (5.4)	10/214 (4.7)	0.38
Caesarian section No. (%)	269/2,236 (12.0)	84/635 (13.2)	25/179 (13.9)	34/242 (14.0)	25/214 (11.7)	0.72

Data are given as number/the total of participants who answered the specific question (%), mean (± Standard Deviation) and median (interquartile range: 25th percentile- 75th percentile). Data given as mean and median are given with the total amount of participants who filled out the specific question (N=). * = Data given as mean. ** = Data given as median. LSC-r score, life stressor checklist – revised calculated score; Education is classified according to the International Standard Classification of Education; GA, Gestational Age.

*^a^Participants with SARS-CoV-2-specific antibodies who filled out the PSS questionnaire were divided into PSS subgroups (low, moderate and high). The p-value represents whether there is a significant difference between the low, moderate and high perceived stress groups*.

#### Postpartum PSS and LSC-r Scores

**Figure 1:**
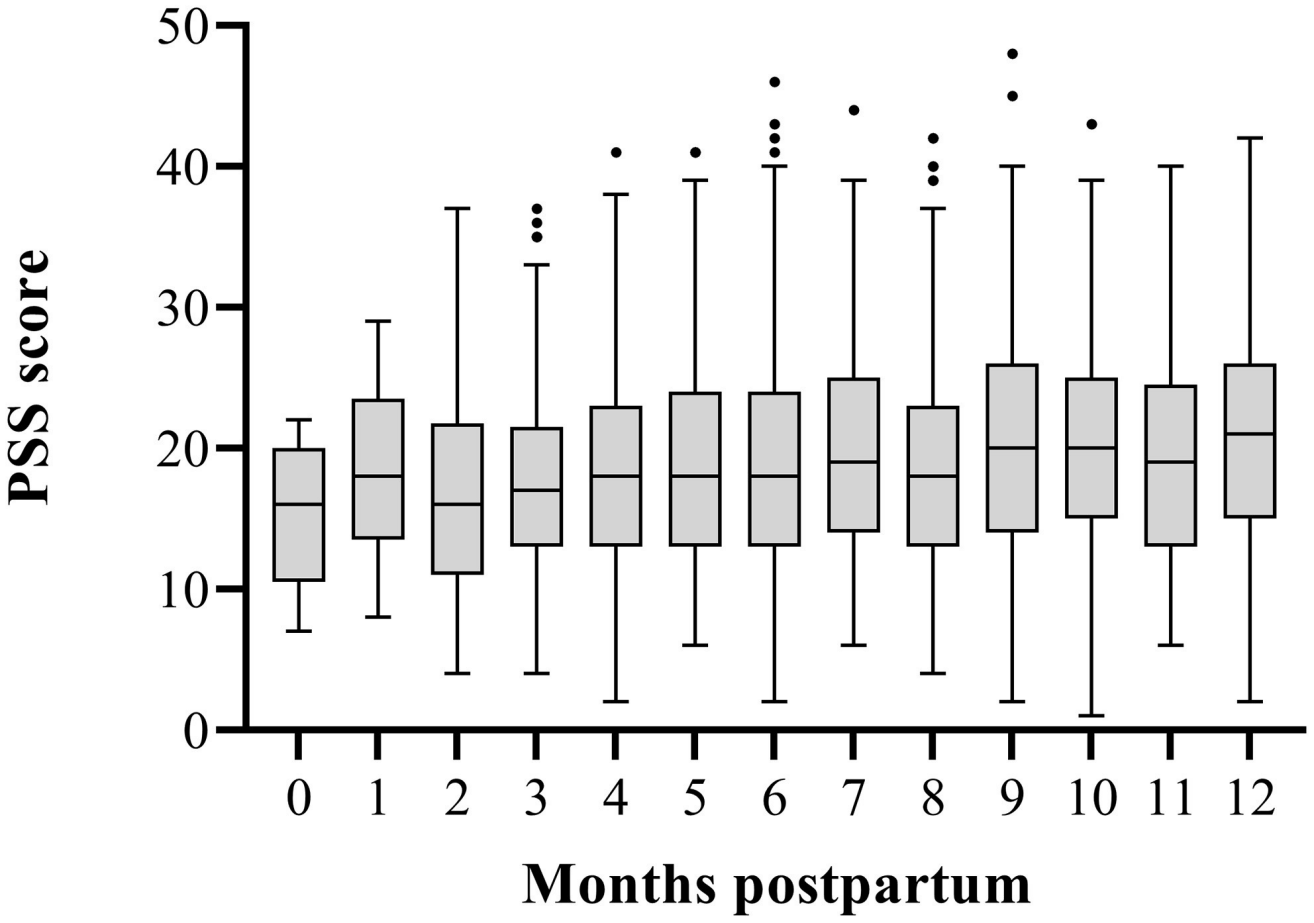
Perceived Stress Scale (PSS) scores up to 12 months postpartum. This figure shows the increase in PSS scores over the first postpartum year. The box represents the interquartile range with median PSS scores. Whiskers present the data range (Q1/Q3 +/−1.5IQR). ·= outlier.

**Figure 2:**
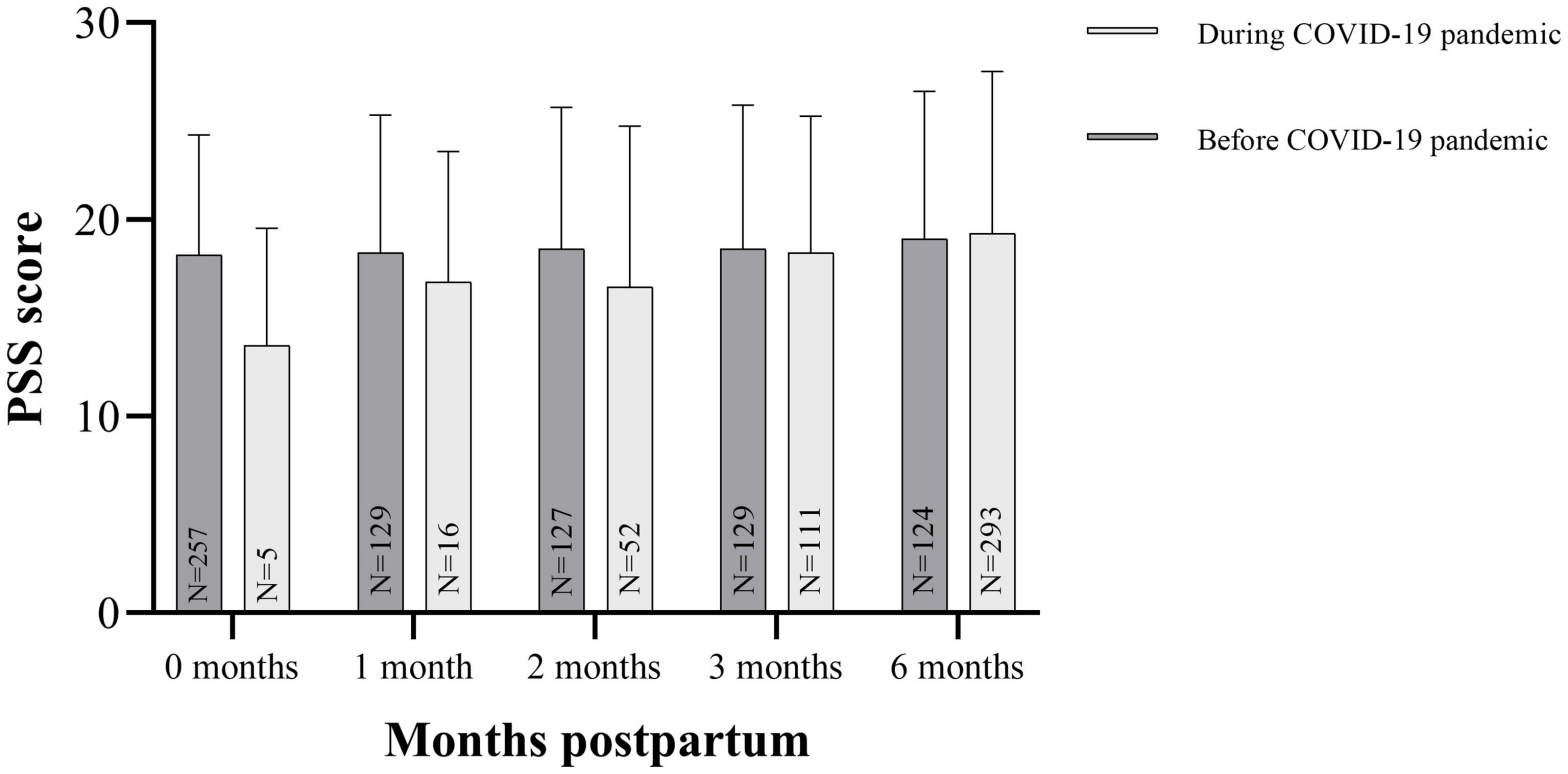
Perceived Stress Scale (PSS) scores during and before the COVID-19 pandemic. In this figure, the PSS scores are displayed as mean (SD) of the specific postpartum group up to six months postpartum. Mean PSS scores of a U.S. cohort before the COVID-19 pandemic are obtained in Paul et al. (34). There were no differences between our study cohort and the pre-pandemic cohort.

The PSS questionnaire was completed by 2,162 participants (94%). These women had a mean PSS score of 19.56 (SD ± 7.97). The PSS scores increased over the first postpartum year [*r* = 0.09, 95% CI: 0.12–0.40, *p* < 0.001, *N* = 1,619 (two-tailed)] (Figure 1). We compared the PSS scores of the women in our cohort to PSS scores in a pre-COVID-19 cohort of lactating women. The mean PSS score in this pre-pandemic cohort of 151 lactating women up to 6 months postpartum was 18.69 (SD ± 0.47) (34). The women up to 6 months postpartum in our cohort had a mean PSS score of 18.41 (SD ± 7.64) (*N* = 494), which did not differ from the pre-pandemic cohort at any time postpartum (mean difference: −0.27, 95% CI: −0.95–0.40, *p* = 0.43) (Figure 2). The LSC-r questionnaire was completed by 2162 participants (94%) and they scored a median of 1.00 (IQR: 0.0–3.0).

### Maternal Stress and *SARS-CoV-2*-Specific Antibodies in Human Milk

#### Baseline Characteristics

Of the total study population, 691 participants tested positive for *SARS-CoV-2*-specific IgA in human milk or IgG in serum. These participants were categorized into subgroups based on their PSS scores: low (*N* = 182), moderate (*N* = 245) and high (*N* = 219) PSS groups. The per subgroup characteristics are depicted in Table 1. Women with high PSS scores had more mental illnesses (*p* < 0.0001), were breastfeeding for a longer time period (*p* = 0.005), smoked more often (*p* = 0.048), and scored higher on the LSC-r questionnaire (*p* < 0.0001).

#### PSS Scores and Maternal SARS-CoV-2-Specific Antibodies

**Figure 3:**
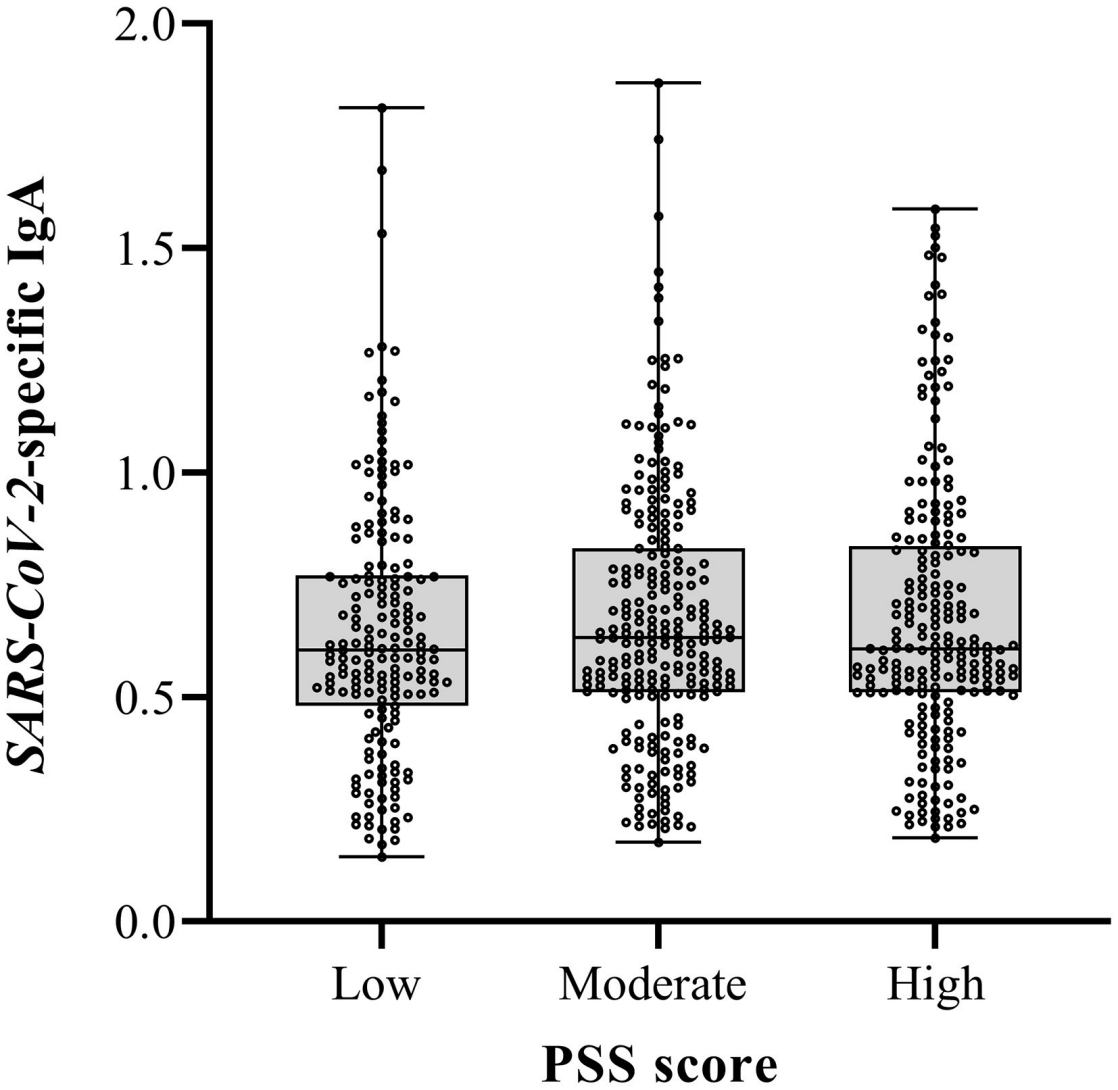
Perceived Stress Scale (PSS) scores and *SARS-CoV-2* specific Immunoglobuline A (IgA) in human milk. The boxes represent the interquartile range with median *SARS-CoV-2* specific IgA in human milk for the different PSS groups. Whiskers present the data range (Q1/Q3 +/−1.5IQR). The dots indicate the individual measurements. No differences in SARS-CoV-2 specific human milk IgA were found between groups.

To compare maternal *SARS-CoV-2*-specific antibodies between the PSS groups, a multiple regression was performed. No differences were observed in *SARS-CoV-2*-specific antibody levels in human milk between the PSS groups in both the unadjusted and adjusted model (Table 2, Figure 3).

**Table 2:** The association between PSS scores and *SARS-CoV-2*-specific antibodies in human milk.

**PSS score subgroups**	**Unadjusted model**		**Adjusted model**	
	**β (95% CI)**	***P*-value**	**β (95% CI)**	***P*-value**
Low - Moderate	1.04 (0.96–1.13)	0.34	1.06 (0.97–1.15)	0.23
Low - High	1.04 (0.96–1.13)	0.35	1.07 (0.98–1.17)	0.15
Moderate - High	1.00 (0.92–1.08)	0.98	1.01 (0.93–1.10)	0.76

*PSS, Perceived Stress Scale*.

*The regression model was adjusted for LSC-r scores, psychological disease, smoking, the age of the child and education level*.

#### LSC-r Scores and Maternal SARS-CoV-2-Specific Antibodies

**Figure 4:**
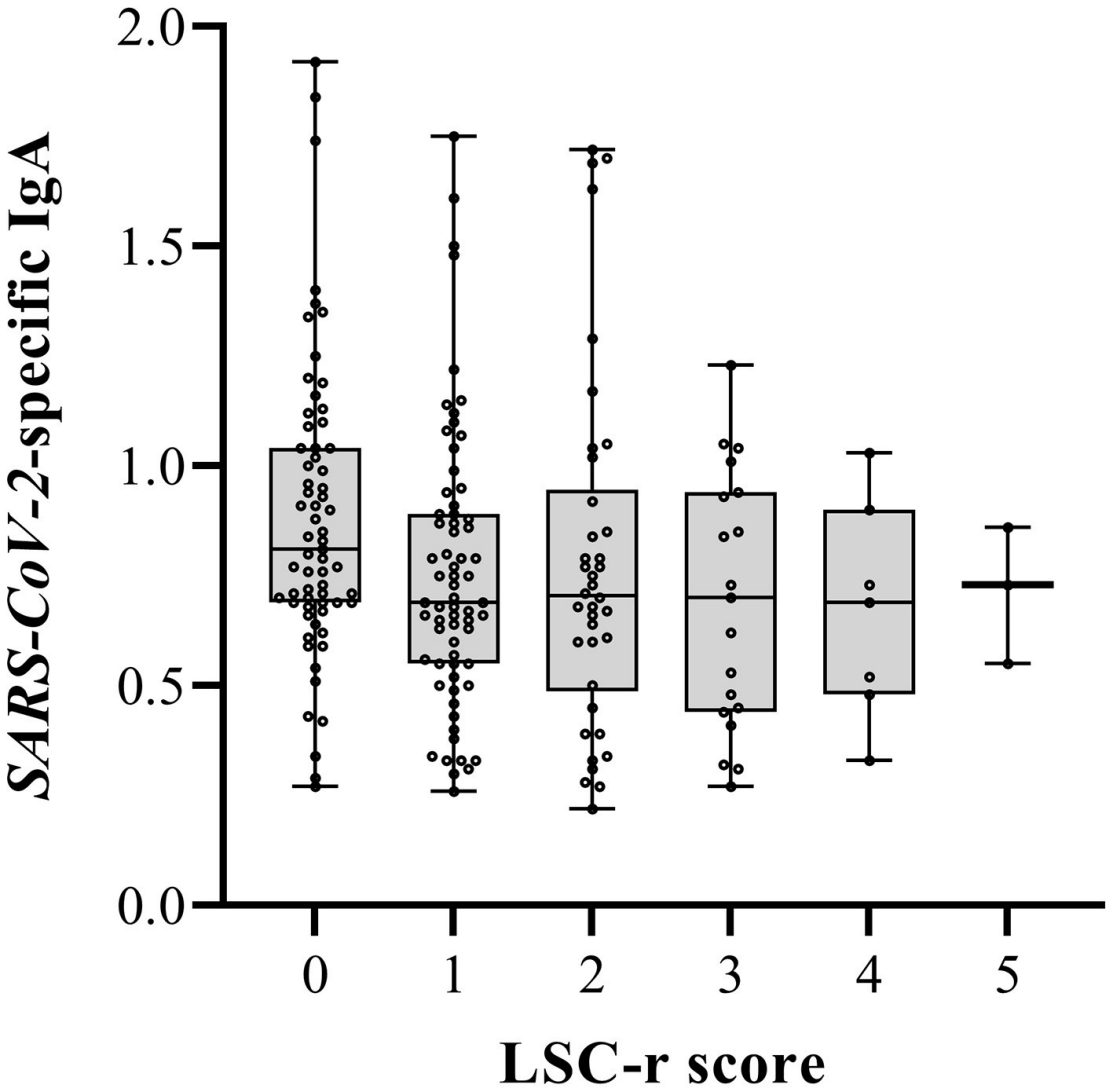
Life Stressor Checklist-revised (LSC-r) scores and *SARS-CoV-2* specific Immunoglobuline A (IgA) in human milk. The boxes represent the interquartile range with median *SARS-CoV-2* specific IgA in human milk for the different LSC-r scores. Whiskers present the data range (Q1/Q3 +/−1.5IQR). The dots indicate the individual measurements. Multiple lineair regression models were used to determine the association between *SARS-CoV-2*-specific IgA in human milk and the LSC-r scores (adjusted *p*-value 0.03).

To investigate the relationship between LSC-r scores and maternal *SARS-CoV-2*-specific antibodies, a multiple regression was performed. After adjustment for covariates, the LSC-r score was negatively associated with IgA in human milk (B = 0.98, 95% CI: 0.96–0.997, *p* = 0.03) (Table 3, Figure 4).

**Table 3:** The association between LSC-r scores and *SARS-CoV-2*-specific antibodies in human milk.

	**Unadjusted model**		**Adjusted model**	
	**β (95% CI)**	***P*-value**	**β (95% CI)**	***P*-value**
LSC-r score	0.98 (0.97–1.00)	0.08	0.98 (0.96–0.997)	0.03

*LSC-r, Life Stressor Checklist – revised*.

*We included age child, sex infant, parity, BMI, age mother to test for potential covariates. The age of the child was considered a confounder and was adjusted for in our model*.

## Discussion

In contrast to our hypothesis, the results of this study suggest that lactating women did not experience higher levels of stress during the COVID-19 pandemic compared to lactating women before the pandemic. Interestingly, maternal lifetime stressors, but not current perceived stress, were negatively associated with human milk antibodies against *SARS-CoV-2*.

Several studies assessed stress levels in lactating women during the COVID-19 pandemic, of which the majority showed that stress and anxiety levels were increased, while some studies showed similar stress levels during and before the pandemic (26, 28, 37–43). The studies that found higher stress levels were carried out at the onset of the pandemic. It could be suggested that the lack of knowledge of the effects of COVID-19 in lactating women and infants at the very beginning of the pandemic caused stress. Considering that our study was conducted 7 months into the pandemic, this could entail that the women who participated in this study had potentially already adapted to the situation and that stress levels were normal again. Moreover, it could be suggested that lactating women did not experience increased stress levels during the pandemic due to other factors, such as a reduction in social and work obligations. For example, working from home results in reduced travel time and spending more time with family (44, 45).

Previous literature on the relationship between stress and human milk antibodies is controversial. Either positive, negative and no associations between maternal stress, anxiety or depression and human milk IgA have been reported (19, 22–25, 46). The before mentioned studies were hampered by their relatively small samples sizes (*n* = 50–119) and differed in type and timing of stress measurement, set up and human milk collection, hampering comparability between studies. Most of the before mentioned literature showed a negative association between maternal stress and human milk antibodies (19, 23, 25, 47). In our study, perceived stress among postpartum women showed no relation with *SARS-CoV-2*-specific antibodies in human milk. However, an inverse association between lifetime stressors and human milk antibodies was observed, also after correcting for possible confounders. This suggests that chronic stress levels may have more pronounced consequences for the maternal immune system compared to current stress levels. Indeed, former research states that chronic stress diminishes the immune response (22, 48–51).

Our study is strengthened by the large sample size, making it possible to identify and adjust for confounding factors. Human milk samples were collected in a standardized way, to minimize collection bias. Moreover, both acute as well as chronic stress was measured. Finally, the study questionnaire was completed by 94% of our study population, which minimizes missing data and improves the reliability and generalizability of our study results. A limitation of our study is that the stress levels were self-reported via questionnaires and that no biological stress measures were included. Moreover, our cohort consisted mostly of highly educated women. It might be that this is not entirely representative for perceived stress levels of all lactating women. In addition, to compare stress levels during the pandemic with pre-pandemic stress levels, our cohort was compared to a pre-pandemic cohort from the United States. Preferably, pre- and during pandemic stress levels should be measured in the same cohort. Finally, *SARS-CoV-2*-specific antibodies may depend on several other factors, including time after infection and severity of symptoms. However, as our sample size is relatively large, we expect that the influence of these factors on our results is minimal.

At this point, we can only speculate what the stress-related changes in human milk antibodies mean for the protection of the breastfed infant. However, as infants drink this milk multiple times a day for a long period, it can be suggested that the protection will be affected. Large sample-sized, population-based studies are needed to address the actual effect of decreased human milk antibody levels on the protection of the breastfed infant from infections. Moreover, future studies should consider adding biological indicators of stress, for example human milk or hair cortisol concentrations, to assess stress levels in lactating women. Lastly, it would be valuable to measure total immunoglobulins and/or other immunological components in human milk to be able to investigate the effects of stress on the total immunological properties of human milk.

## Conclusion

The results of this study demonstrated that lactating women in the Netherlands did not experience higher perceived stress levels seven months into the COVID-19 pandemic compared to stress levels of lactating women prior to the COVID-19 pandemic. Moreover, lifetime stress was associated with reduced *SARS-CoV-2*-specific antibodies in human milk, while current perceived stress was not. Our findings emphasize the importance of psychological well-being of lactating women and the need to identify and guide (expecting) mothers with high chronic stress levels.

## Data Availability Statement

The raw data supporting the conclusions of this article will be made available by the authors, without undue reservation.

## Ethics Statement

The studies involving human participants were reviewed and approved by METc VUmc. The patients/participants provided their written informed consent to participate in this study.

## Author Contributions

HJ and BK had full access to all of the data in the study and take responsibility for the integrity of the data and the accuracy of the data analysis. Concept and design: HJ, ER, AK, JG, and BK. Acquisition, analysis, or interpretation of data: HJ, ER, MG, AK, JG, and BK. Drafting of the manuscript: HJ, ER, and BK. Critical manuscript revision for intellectual content: MG, AK, JG, and BK. Statistical analysis: HJ, ER, and BK. Obtained funding: MG, JG, and BK. Administrative, technical, or material support: HJ, ER, MG, JG, and BK. Supervision: JG and BK. All authors contributed to the article and approved the submitted version.

## Funding

This study was funded by Stichting Steun Emma Kinderziekenhuis. The funder did not have any influence on the data collection or interpretation.

## Conflict of Interest

JG was the founder and director of the Dutch National Human Milk Bank and a member of the National Health Council and has been a member of the National Breastfeeding Council from March 2010 to March 2020.

The remaining authors declare that the research was conducted in the absence of any commercial or financial relationships that could be construed as a potential conflict of interest.

## Publisher's Note

All claims expressed in this article are solely those of the authors and do not necessarily represent those of their affiliated organizations, or those of the publisher, the editors and the reviewers. Any product that may be evaluated in this article, or claim that may be made by its manufacturer, is not guaranteed or endorsed by the publisher.
